# Data in support of fumosorinone, a novel PTP1B inhibitor, activates insulin signaling in insulin-resistance HepG2 cells and shows anti-diabetic effect in diabetic KKAy mice

**DOI:** 10.1016/j.dib.2015.03.006

**Published:** 2015-04-01

**Authors:** Du-Qiang Luo, Zhi-Qin Liu, Ting Liu, Chuan Chen, Ming-Yan Li, Zi-Yu Wang, Ruo-song Chen, Gui-xiang Wei, Xiao-yi Wang

**Affiliations:** a College of Life Sciences, Key Laboratory of Medicinal Chemistry and Molecular Diagnosis of Ministry of Education, Hebei University, Baoding 071002, PR China; b College of Pharmaceutical Sciences, key laboratory of pharmaceutical quality control of Hebei province, Hebei University, Baoding 071002, PR China

## Abstract

This data article contains data related to the research article entitled “Fumosorinone, a novel PTP1B inhibitor, activates insulin signaling in insulin-resistance HepG2 cells and shows anti-diabetic effect in diabetic KKAy mice” in the Toxicology and Applied Pharmacology [1]. Fumosorinone (FU) is a new inhibitor of protein phosphatase 1B inhibitor, which was isolated from insect pathogenic fungi Isaria fumosorosea. FU was found to inhibit PTP1B activity in our previous study [2]. PTP1B is the physiological antagonist of the insulin signalling pathway. Inhibition of PTP 1B may increase insulin sensitivity [3]. PTP1B has been considered promising as an insulin-sensitive drug target for the prevention and the treatment of insulin-based diseases [4]. We determined the effect of FU on the glucose consumption of IR HepG2 cells. FU caused significant enhancement in glucose consumption by insulin-resistant HepG2 cells compared with control cells.

## 1 Specifications table

**Table:** 

Subject area	Pharmacy
More specific subject area	Antidiabetic effect of natural products
Type of data	Figure
How data was acquired	The cell plates were read on a Synergy HT microplate reader (PerkinElmer, MA, USA) at 550 nm.
Data format	Analyzed
Experimental factors	The IR HepG2 cells were exposed to FU (0-20μM) or Rosiglitazone (10μM RGZ) for 24 h.
Experimental features	Glucose consumption in IR HepG2 was assessed.
Data source location	Baoding, China
Data accessibility	Data is provided in the article.

## 2 Value of the data

• The glucose consumption was significantly increased by 1.25- 20μM FU treatment.
• The data demonstrated the FU can improve insulin resistance in vitro.
• The data provided tested doses for further study in the research article.

## 3 Experimental design, materials and methods

### 3.1 Cell culture

Hepatocellular carcinoma cells (HepG2 cells) possess the same bioactivity as normal hepatic cells, which are valuable for investigating liver-associated functions and stable during many passages [5]. HepG2 cells were purchased from National Platform of Experimental Cell Resources for Sci-Tech (Beijing, China). HepG2 cells were routinely grown in Minimum Essential Medium (MEM) supplemented with 10% (v/v) fetal bovine serum, streptomycin (100μg/mL), and penicillin (100U/mL), in a humidified atmosphere of 95 % air–5% CO_2_ at 37 °C.

### 3.2 Glucose consumption assay

**Fig. 1:**
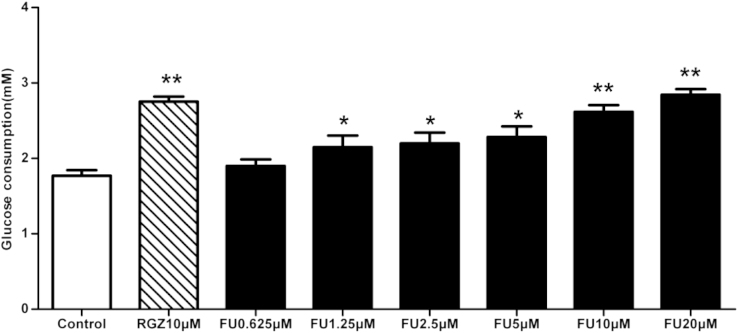
Effect of FU on glucose consumption in insulin-resistant HepG2 cells. The insulin-resistant HepG2 cells were treated with different concentration FU or RGZ for 24h, and then incubated with 100nM insulin for 30min. After this incubation, glucose content in the culture medium was measured by glucose oxidase method. Values are the mean ± SD of three independent experiments. ^⁎^P<0.05, ^⁎⁎^P<0.01 compared with control.

The establishment of an insulin-resistant HepG2 cell model and glucose consumption were performed according to the reported method [6,7] with a slight modification. Briefly, HepG2 cells were cultured in 96-well cluster plates. After reaching confluence, the cells were treated with 10^−6^ mol/L insulin for 24 h to induce insulin resistance, then add in different concentrations FU (0-20μM) or Rosiglitazone (RGZ, 10μM, was purchased from TCI, Tokyo, Japan) [8] and incubated for 24 h, and then incubated with 100nM insulin (was purchased from Sigma-Aldrich, USA) for 30 min. After this incubation, glucose content in the culture medium was measured by glucose oxidase method (The Glucose Analysis Kit was purchased from Applygen Technologies Inc. Beijing, China). The amount of glucose consumed was calculated by measuring glucose concentrations of blank wells and subtracting the remaining glucose in cell-plated wells [6]. As shown in Fig. 1. The glucose consumption was significantly increased by 1.25, 2.5, 5, 10 and 20μM FU treatment for 24 h. The effect of FU at a concentration of 10 and 20μM on glucose consumption was similar to that of RGZ.

## 4 Statistical analysis

The data were expressed as mean ±SD. Differences between the groups were compared by one-way analysis of variance (ANOVA), followed by a Dunnett׳s multiple comparison test. *P*<0.05 was considered to be statistically significant differences between groups.

## Conflict of interest statement

The authors declare that they have no conflict of interest associated with this manuscript.
